# The Royal Hospital for Incurables

**Published:** 1906-09-29

**Authors:** 


### HOSPITAL ADMINISTRATION.

### CONSTRUCTION AND ECONOMICS.

### THE ROYAL HOSPITAL FOR INCURABLES.

### ITS GOOD AND BAD POINTS.

A Letter from the President.

In announcing the appointment of the new Secretary in our issue of August 4 last, we pointed out "that a hospital for incurables is a very difficult establishment to administer justly and efficiently," and stated that "we had always felt that the Royal Hospital for Incurables at Putney needed a strong hand to overhaul the whole administration, to soften and modernise it, and so to add greatly to the efficiency and popularity of this institution." On the 20th instant we received a letter from Mr. W. D. Newton, the secretary, stating that he was desired by Lord Northampton to ask that the following letter might be published in The Hospital. The Marquis of Northampton, who is the President of the hospital, writes as follows from Loch Luichart, Ross-shire, under date of the 17th instant:—

My attention has been called to a paragraph in The Hospital of August 4 on the subject of the Royal Hospital for Incurables. The final sentence seems to be a reflection on the administration of the hospital, and a suggestion that the secretary who has just been appointed will be able to put things right which have hitherto been wrong. The hospital is managed by a very efficient Committee, and as President I can vouch for the fact that nothing can be done which has not been done "to soften and modernise it," or to make it more efficient and more popular. We have had the benefit of the services of a very zealous and able Secretary, whose resignation emanated entirely from himself; and I am at a complete loss to understand what can have been the object of a misleading and unfair statement.

It will be seen that no reference was made to the late secretary, even by implication, in our statement, nor had we that gentleman or his office in any way in mind. Indeed, before Lord Northampton's letter reached us we had had a representation from another source to the effect that the office of the secretary being situated in the City, and the responsibility of his duties in looking after a revenue of tens of thousands of pounds, made it impossible for him to attend to the administration of the home at Putney. We are in agreement with this view and in referring to the need of "a strong hand to overhaul the whole administration, to soften and modernise it," we had in mind the recommendations of the House of Lords Commission on Hospitals, and the memorandum and recommendations of the Earl of Aberdeen dated July 1893, who was chairman of the committee of inquiry held in that year. The secretary could probably only use his influence in favour of one great reform in the general administration of the charity, namely the abolition of the voting system. This entails very real hardship and cruelty upon the unfortunate people who need the shelter of an institution of this kind, as we proved by the facts given in our issue of September 19, 1903, page 441.

"The strong hand needed to overhaul the whole administration, to soften and modernise it," would necessarily abolish the voting system, and would at the same time secure the adoption of Lord Aberdeen's recommendations which included (1) the importing of a new feature into the system of management, namely the appointment of a resident medical officer or superintendent; (2) the election of a certain number of ladies upon the board or committee of management. Neither of these three important reforms has been introduced into the management of the Royal Hospital for Incurables at Putney, although they were strongly advocated, and convincing reasons were produced at the inquiry which showed the importance and necessity that the changes should be made without delay in the best interests of the hospital and its inmates. We hope therefore that the time is approaching, if it has not already come, when, with the aid of the president, Lord Northampton, it may be possible to introduce these necessary reforms and so to make the Royal Hospital for Incurables what it in fact ought to be, the best administered and most efficient hospital for incurables in the world.

We are very anxious not to be misunderstood. We desire to give full credit for the improvements which have taken place in the last few years since the appointment of the present matron. A recent inspection of the institution shows that under her the comfort and well-being of the patients have greatly improved. That marked attention is paid to making the wards and rooms comfortable and attractive, and to secure that in the service of meals little details are not overlooked which add materially to comfort. In the matter of food and the provision of certain comforts and even luxuries the patients at the present time are exceptionally well cared for at Putney.

Another and great improvement which is most marked is the higher tone which seems to prevail throughout the establishment. At the present time the patients appear to be entirely at ease. They are on the best of terms with their nurses and attendants, and do not hesitate to ask readily for anything which they may require from time to time. A spirit of cheerfulness seems to dominate the patients, which is a good sign, and may be taken to show that they regard the hospital as a home for life, where they may feel independent and so are good humoured and cheerful. The effect of securing that the matron and the superintendent nurses are all trained has been very good in a hygienic sense, but we are of opinion that the attendants who are untrained should not be called assistant nurses, but attendants simply, as they have no proper claim to the title of nurse. There can be no question that the retirement of the three principal officials some years ago led to many important changes, which must have added immeasurably to the comfort and well-being, as well as to the happiness, of the inmates. This good result is everywhere manifested to-day throughout the institution. The matron gives the visitor the impression of being a thoroughly capable administrator; the patients are undoubtedly well cared for, happy and at ease, and the care and handling of chronic cases, which is now in the hands of attendants who are under the control and supervision of trained nurses, has yielded important results for good. All this is satisfactory, but the present is a progressive age, and the Royal Hospital for Incurables will never be the great institution it can and ought to be until it has abolished the voting system, admitted women to the board of management, and elected a competent medical superintendent to take charge as the responsible head of the whole institution.

